# The crystal structures of PKG Iβ (92-227) with cGMP and cAMP reveal the molecular details of cyclic-nucleotide binding

**DOI:** 10.1186/1471-2210-11-S1-O14

**Published:** 2011-08-01

**Authors:** Jeong Joo Kim, Darren E Casteel, Gilbert Huang, Taek Hun Kwon, Ronnie Kuo Ren, Peter Zwart, Jeffrey J Headd, Nicholas Gene Brown, Dar-Chone Chow, Timothy Palzkill, Choel Kim

**Affiliations:** 1 Department of Pharmacology, Baylor College of Medicine, One Baylor Plaza, Houston, TX 77030 USA; 2 Department of Medicine, University of California, San Diego, 9500 Gilman Dr. La Jolla, CA 92093, USA; 3 The Verna and Marrs McLean Department of Biochemistry and Molecular Biology, Baylor College of Medicine, One Baylor Plaza, Houston, TX 77030, USA; 4 Rice University, 6100 Main Street, Houston, TX 77005-1827, USA; 5 The Berkeley Center for Structural Biology, Lawrence Berkeley National Laboratory, Berkeley, California 94720, USA; 6 Department of Molecular Virology and Microbiology, Baylor College of Medicine, One Baylor Plaza, Houston, TX 77030, USA

## Background

Cyclic GMP is a crucial second messenger that translates extracellular signals into a variety of cellular responses. As a central mediator of the Nitric Oxide-cGMP signalling cascade, which regulates vascular tone, platelet aggregation, nociception and hipocampal/cerebellar learning, Cyclic GMP-dependent protein kinases (PKGs) represents an important drug target for treating hypertensive diseases and erectile dysfunction.

The fidelity of the NO-cGMP signalling pathway is largely dependent on PKG’s ability to selectively bind cGMP over cAMP. Although both cGMP and cAMP bind and activate PKG, cGMP preferentially activates PKG 60-100 fold better than cAMP; yet, little is known about the molecular features required for the cGMP selectivity of PKG. We have investigated the mechanism of cyclic nucleotide binding to PKG by determining crystal structures of the amino-terminal cyclic nucleotide-binding domain (CNBD-A) of human PKG I bound to either cGMP or cAMP. We also determined the structure of CNBD-A in the absence of bound nucleotide.

## Results

The crystal structures of CNBD-A with bound cAMP or cGMP reveal that cAMP binds in either *syn* or *anti* configurations whereas cGMP binds only in a *syn* configuration, with a conserved threonine residue anchoring both cyclic phosphate and guanine moieties. The structure of CNBD-A in the absence of bound cyclic nucleotide was similar to that of the cyclic nucleotide bound structures. Surprisingly, isothermal titration calorimetry experiments demonstrated that CNBD-A binds both cGMP and cAMP with a relatively high affinity, showing an approximately two-fold preference for cGMP.

**Table 1:** Data and refinement statistics

Data set	cGMP bound	cAMP bound	Partial APO
Space group	*P6*_2_*22*	*P6*_2_*22*	*P4*_3_
Cell constants (Å)	a=b=107, c=171 α=β=90.0, γ=120	a=b=107, c=169 α=β=90.0, γ=120	a=b=62.6, c=202 α=β=γ=90.0
Wavelength (Å)	1.0	1.0	1.0
Resolution (Å)	50 – 2.9	50 – 2.49	45-2.75
Total/unique reflections	402498/13503	293611/20607	80424/19782
Average redundancy	29.8(20.2)	13.8(14.2)	4.1(4.1)
Completeness (%)	100(100)	98.7(99.6)	100(99.6)
<I>/<σ_I_>	21.2(2.10)	43.5(5.62)	31.2 (2.39)
R_sym_^◊^ (%)	13.5(n/a)	10.1(42.4)	5.9(46.4)
R_work_ (%)	20.4	20.6	18.0
R_free_^¶^ (%)	26.0	23.0	25.1
Overall B value( Å^2^)	73.4	46.6	94.4
Rmsd bond length (Å)	0.010	0.014	0.005
Rmsd bond angle(°)	1.42	1.274	0.942

^◊^*R*_sym_ = ∑*_h_*∑*_i_*|*I*(*h*) - *I*(*h*)*_i_*|*I*∑*_h_*∑*_i_I*(*h*)*_i_*, where *I*(*h*) is the mean intensity after rejections.

^§^Numbers in parentheses correspond to the highest resolution shell of data, which were 2.98 to 2.90 for the cGMP, 2.53 to 2.49 Å for the cAMP and 2.85 to 2.75Å for APO.

^||^*R*_work_ = ∑*_h_*||*F*_obs_(*h*)| -|*F*_calc_(*h*)||*I*∑*_h_*|*F*_obs_(*h*)|; no *I*/σ cutoff was used during refinement.

^¶^ 5.0% of the observed intensities was excluded from refinement for cross validation purposes.

**Figure 1:**
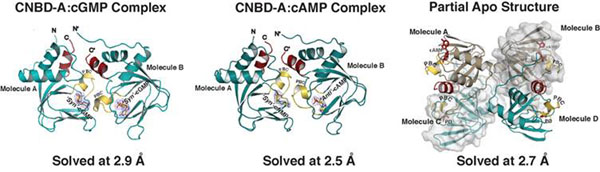


Overall structure of the PKG Iβ CNBD-A:cGMP complex showing on the left, the PKG Iβ CGBD-A:cAMP complex in the middle, and the partial apo on the right. All three crystals contained more than one molecule per unit cell, which enable us to sample different modes of interaction with cyclic nucleotides. The phosphate binding cassette (PBC) is shown in yellow, the αB helix in red and N- and C-termini are labeled. For the cGMP and cAMP complexes, bound cyclic nucleotides are shown with the *Fo-Fc* omit map electron density.

**Figure 2:**
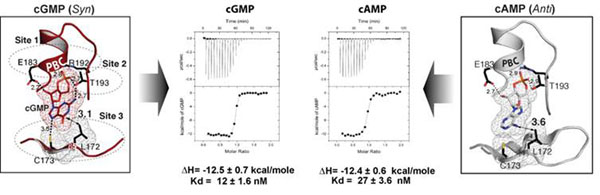


Cyclic Nucleotides interacting with the cGMP pocket. Both cGMP and cAMP bond in the cGMP binding pocket are shown on the far left and right and their Isothermal titration calorimetry data binding to the PKG Iβ CNBD-A shown in the middle. The cGMP-binding site is marked with three different sites: the short P-helix together with conserved glutamate and arginine residues at the PBC which captures the sugar phosphate (Site 1); a key residue, Thr^193^ at the end of PBC that bridges the cyclic phosphate to the guanine ring (Site 2); and the β5-strand that provides a unique docking site for the guanine ring (Site 3). Unlike cGMP, cAMP binds in two different configurations, *anti* in one molecule (shown on the far right panel) and *syn* in the other with different sets of contacts. Although the sugar phosphates share the same set of contacts with the protein at site 1, each purine ring of cAMP shows different contacts with the protein at sites 2 and 3The calorimetric measurements for cAMP and of cGMP binding to PKG Iβ (92-227) were carried out using a VP-ITC calorimeter (MicroCal LLC, Northampton, MA).

## Conclusion

Our findings suggest that CNBD-A binds cGMP in the *syn* conformation through its interaction with Thr193 and an unusual cis-peptide forming residues Leu172 and Cys173. Although these studies provide the first structural insights into cyclic nucleotide binding to PKG, our ITC results show only a two-fold preference for cGMP, indicating that other domains are required for the previously reported cyclic nucleotide selectivity.
